# Pregnancy Rate after First Intra Cytoplasmic Sperm Injection- *In Vitro* Fertilisation Cycle in Patients with Endometrioma with or without Deep Infiltrating Endometriosis 

**Published:** 2013-09-18

**Authors:** Anne Oppenheimer, Marcos Ballester, Emmanuelle Mathieu d’Argent, Karine Morcel, Jean-Marie Antoine, Emile Daraï

**Affiliations:** 1 Department of Gynecology-Obstetrics, Tenon Hospital, GRC-UPMC 6 (C3E), Pierre et Marie Curie Paris 6 University, Paris, France; 2 Department of Gynecology-Obstetrics, Rennes Hospital, Paris, France

**Keywords:** Endometrioma, Assisted Reproductive Technology, Endometriosis, Probabilistic Model

## Abstract

**Background:**

To evaluate the impact of the association of endometrioma with or without deep infiltrating endometriosis (DIE) after a first intra cytoplasmic sperm injection- in vitro fertilization (ICSI-IVF) cycle on pregnancy rate.

**Materials and Methods:**

In this retrospective study, women with endometrioma who underwent a first ICSI-IVF cycle from January 2007 to June 2010 were reviewed for pregnancy rate. The main outcome measure was the clinical pregnancy rate. A multiple logistic regression (MLR) was performed; including all variables that were correlated to the conception rate. Only independent factors of pregnancy rate were included in a Recursive Partitioning (RP) model.

**Results:**

The study population consisted of 104 patients (37 without DIE and 67 patients with associated DIE). Using multivariable analysis, a lower pregnancy rate was associated with the presence of DIE (OR=0.24 (95% CI: 0.085-0.7); p=0.009) and the use of ICSI (OR=0.23 (95% CI: 0.07-0.8); p=0.02). A higher pregnancy rate was associated with an anti-mullerian hormone (AMH) serum level over 1 ng/ml (OR=4.3 (95% CI: 1.1-19); p=0.049). A RP was built to predict pregnancy rate with good calibration [ROC AUC (95% CI) of 0.70 (0.65-0.75)].

**Conclusion:**

Our data support that DIE associated with endometrioma in infertile patients has a negative impact on pregnancy rate after first ICSI-IVF cycle. Furthermore, our predictive model gives couples better information about the likelihood of conceiving.

## Introduction

Three types of endometriosis have been described: peritoneal endometriosis, ovarian endometriosis (known as endometrioma) and deep infiltrating endometriosis (DIE) (1). These are often associated with endometriosis-related infertility and the extent and location affect the chances of pregnancy in women (2). While the American Society of Reproductive Medicine (ASRM) classification is a useful tool to compare studies, its relevance in predicting fertility outcomes according to endometriosis stage is debatable (3). Moreover, the ASRM classification does not take into account the presence of DIE (2).

Despite the limits of the ASRM classification, two randomized studies (4, 5) and a meta-analysis (6) have demonstrated the positive impact of removing endometriotic lesions in patients with I-II ASRM stages on spontaneous fertility. A more recent meta-analysis has demonstrated the absence of a positive impact of removing endometriomas before *in vitro* fertilization (IVF) on fertility outcomes (7). Moreover, several studies have underlined the negative effect of cystectomy for endometriomas on ovarian reserve, evaluated by anti-mullerian hormone (AMH) serum level or antral follicle count and response to IVF stimulation, particularly in patients with bilateral cysts (8, 9). Some controversy remains over the impact of DIE on fertility. Stepniewska et al. (10) suggested that the removal of DIE was associated with enhancement of both spontaneous pregnancy and increased fertility results in assisted reproductive therapy (ART). Moreover, this study revealed that incomplete resection of DIE was associated with a lower pregnancy rate compared with patients undergoing complete removal. Barri et al. (11) also demonstrated that the best option for infertile patients with endometriosis, depending on their age, was the combination of surgery and IVF. However, Mathieu d’Argent et al. (12) reported similar ICSI-IVF pregnancy rates in patients with DIE and colorectal involvement as in those with tubal or male infertility. This raises the question of whether surgery, which exposes patients to the risk of severe complications, is a legitimate option to enhance fertility outcomes in ART. However, none of these authors were able to demonstrate whether the association of endometrioma with DIE, a common occurrence, has an impact on IVF results.

The aims of this study were therefore to evaluate the impact on pregnancy rate of endometrioma associated with DIE after a first ICSI-IVF cycle and to evaluate determinant factors to establish a pragmatic approach.

## Materials and Methods

We retrospectively identified 104 women with endometrioma who had undergone ICSI-IVF treatment after at least 1 year of infertility in the Department of Gynecology-Obstetrics at Tenon hospital (France) from January 2007 to June 2010. The investigation of fertility included a hormonal blood test in the third day of the cycle [serum level measurements of estradiol (E2), follicle stimulating hormone (FSH), inhibin B and anti-mullerian hormone (AMH)], a hysterosalpingography, transvaginal sonography and semen analysis for the partner. The diagnosis of endometriosis was made with physical examination, transvaginal sonography and magnetic resonance imaging (MRI) using previously published imaging criteria (13). DIE was diagnosed with physical examination when lesions on the posterior vaginal fornix were found or when we identified some infiltration or nodule on the torus uterinus or uterosacral ligaments. With the transvaginal sonography, DIE was diagnosed when one of these structures were involved: vagina, uterosacral ligament, rectovaginal septum, rectosigmoid colon and bladder. The diagnosis of DIE was done on abnormal hypoechoic linear thickening and nodules. With MRI, the diagnosis of DIE was done on the combined presence of signal abnormalities on the same structure mentioned before.

The patients were divided into two groups; the endometrioma group (37 women) consisting of patients with proved endometrioma without DIE and the endometrioma-DIE group (67 women) consisting of patients with endometrioma and DIE.

Three different forms of down regulation were used: a long gonadotropin-releasing hormone (GnRH) agonist, a short agonist or an antagonist protocol. Ovarian stimulation was done with doses of recombinant FSH between 75 and 450 IU/d depending on patient age, body mass index (BMI), antral follicle count (AFC), AMH, size and number of follicles, and E2 levels. This stimulation was begun once pituitary desensitization (E2 level <50 pg/mL) had been achieved. Transvaginal oocyte retrieval was scheduled 35-36 hours after hCG injection and embryo transfer (ET) was performed 2-3 days later. On day 2, individually cultured embryos were evaluated on the basis of the number of blastomeres, blastomere size, fragmentation rate and presence of multinucleated blastomeres (14). The top quality embryos were defined as having four regular blastomeres with <20% fragmentation. The luteal phase was supported by vaginal administration of micronized P (400 mg/d) from the day of ovarian puncture to the day of the pregnancy test. Pregnancies were diagnosed by an increasing concentration of serum β-human chorionic gonadotropin (β-hCG) which was tested 14 days after ET. Clinical pregnancies were confirmed by the presence of a gestational sac on vaginal ultrasound examination during the fifth week.

For embryo transfer, a soft catheter was used which is inserted through the cervical canal into the uterine cavity. Ultrasound guidance and anesthesia was not required.

### Statistical analysis

Univariate analysis was performed using Student’s t test or Wilcoxon test for continuous variables and Chi-square test or Fisher’s exact test for qualitative variables. We tested epidemiological, biological and radiological characteristics in a multivariate analysis for association with pregnancy rate. A p value of less than 0.05 was considered significant.

Recursive partitioning (RP) was used to determine cut-offs for each variable predicting an improvement in pregnancy rate. RP is a technique which can be applied to examine large datasets to uncover hidden patterns within the data and to elucidate statistically significant sub-groupings within the data. RP is non-parametric in nature, imposing no a priori restrictions on the distributional forms of the predictor variables. The central result is a simple and intuitive RP algorithm. At each step, the RP program determines for each variable cut-points that optimally separate patients into homogeneous groups. A multiple logistic regression (MLR) was performed; including all variables that were correlated to the conception rate. Only independent factors of pregnancy rate were included in a RP model. All analyses were performed using the R package with the Verification, Design, Hmisc, DiagnosisMed, ROCR and Presence Absence libraries.

### Ethical considerations

All the patients gave informed consent to participate in the study. The protocol was approved by the Ethics Committee of the Collège National des Gynécologues et Obstétriciens Français (CNGOF).

## Results

One hundred and four women with endometrioma and proved infertility who had undergone ICSI-IVF cycles were included with only the first cycles being analyzed. The epidemiological characteristics of the whole population are summarized in table 1. The median age of the population study was 32 years and the median BMI was 22.4 kg/m^2^. The median duration of infertility was 3 years.

**Table 1:** Epidemiological characteristics of the 104 patients with endometrioma with or without DIE


Characteristics	Patients Number n=104

**Age (Y) (median , range)**	32 (24-42)
**BMI (kg/m²) (median, range)**	22.4 (17.3-35.4)
**Patients smoking, N (%)**	20 (23.8%)
**Duration of prior infertility (Y), median (range)**	3 (1-9)
**Associated male infertility, N (%)**	43 (41.3%)
**Associated tubal infertility, N (%)**	56 (53.8%)
**Number of endometriomas, median (range)**	2 (1-6)
**Unilateral endometriomas, N (%)**	58 (55.7%)
**Bilateral endometriomas, N (%)**	46 (44.3%)
**Median size of the largest endometrioma (mm) (range)**	33 (10-100)
**Patients with associated DIE, N (%)**	67 (64.4%)
**Patients with prior surgery for endometriosis, N (%)**	72 (69.2%)


Endometriomas were unilateral and associated with DIE in 55.7 and 64.4% of cases respectively. The median number of endometriomas was two and the median size of the largest endometrioma was 33 mm. The proportion of patients with a major endometrioma measuring less than 3 cm, between 3-5 cm and more than 5 cm was 36.5, 51 and 12.5% respectively. Before the ICSI-IVF cycle, AMH serum levels were 4 ng/ml in women with an endometrioma diameter size lower than 3 cm or between 3-5 cm and 3 ng/ml in those with an endometrioma diameter size over 5 cm. No difference in AMH serum levels according to endometrioma sizes was observed. AMH serum levels in patients with or without prior surgery for endometrioma were 2.7 ng/ml and 3.9 ng/ml respectively (p=0.2) and in those with or without prior surgery for DIE were 2.9 and 3 ng/ml respectively (p=0.9).

The epidemiological characteristics of patients with endometrioma with or without DIE are summarized in table 2. No difference in the median age, smoking, duration of infertility or rate of associated tubal and male infertility was found. BMI was higher in the group of patients with endometrioma and DIE (p=0.01). AMH serum levels in patients with endometrioma with or without DIE were 3.2 and 3.4 ng/ml respectively. No difference in AMH serum levels was found between the groups.

**Table 2:** Comparison of epidemiological characteristics of patients with endometrioma with or without DIE


	Patients with endometrioma and DIE n=67	Patients with endometrioma without DIE n=37	P value

**Age (Y), median (range)**	32 (24-42)	33 (24-41)	0.2
**BMI (kg/m^2^), median (range)**	23.12 (17.2-35.4)	21.3 (17.9-29.9)	0.01
**Patients smoking, N (%)**	12 (17.9%)	8 (21.6%)	0.84
**Duration of prior infertility (Y), median (range)**	3 (1-9)	4 (1-7)	0.25
**Associated tubal infertility, N (%)**	40 (59.7%)	16 (43.2%)	0.16
**Associated male infertility, N (%)**	28 (41.8%)	15 (40.5%)	0.93
**Failure of IUI **	7 (10.5%)	15 (40.5%)	0.0008


The pre ICSI-IVF biological characteristics and responses to hormonal ovarian stimulation of the patients with endometrioma with or without DIE are summarized in table 3. No differences in the AMH, inhibin B, E2, AFC, number of ICSI or IVF procedures, types of ovarian stimulation, total dose of gonadotrophin used, number of mature follicles >14 mm, total number of oocytes retrieved, total number of day-2 fresh embryos, number of top day-2 fresh embryos, thickness of endometrium, number of top day-2 embryos transferred and number of embryos cryopreserved were found between the groups. An association between the requirement for ICSI and male infertility was observed (p<0.0001).

Comparison of epidemiological characteristics of patients who conceived and those who did not is given in table 4. Using univariable analysis, the number of patients who conceived was lower in the group of patients with endometrioma and DIE (in the group of patients with endometrioma and DIE: patients who conceived n=22 (51.1%) vs. patients who did not conceive n=45 (73.8%); p=0.03).

**Table 3:** Biological characteristics and responses to ovarian stimulation of patients with endometriomas with or without DIE


	Patients with endometrioma and DIE n=67	Patients with endometrioma without DIE n=37	P value

**AMH serum level (ng/ml), median (range)**	2.8 (0.2-12.4)	2.5 (0.5-14.2)	0.6
**Day 3 Inhibin B (IU/ml), median (range)**	56 (15-125)	49 (15-278)	0.9
**Day 3 E2 (pg/ml), median (range)**	1765 (295-5460)	1802 (520-4185)	0.5
**Total AFC, median (range)**	12 (2-60)	12 (2-28)	0.2
**Type of ART**			
IVF	46	22	0.46
ICSI	21	15	
**Stimulation protocol**			
Antagonist	2	3	0.23
Short agonist	12	3	
Long agonist	53	30	
**Dose of gonadotrophin (UI), mean (range)**	2400 (1100-7650)	2250 (1350-7200)	0.76
**Number of follicles >14 mm **	8 (1-21)	7 (2-16)	0.65
**Number of oocytes retrieved, mean (range)**	8 (1-26)	8 (2-19)	0.24
**Number of day 2 fresh embryos, mean (range)**	4 (0-16)	5 (0-14)	0.29
**Number of day 2 top fresh embryos, mean (range)**	1 (0-8)	1 (0-4)	0.48
**Endometrial thickness (mm), mean (range)**	10 (5-27)	10 (7-16)	0.74
**Number of top day 2 fresh embryos transferred, mean (range)**	1 (0-2)	1 (0-2)	0.93
**Number of day 2 frozen embryos transferred, mean (range)**	1 (0-12)	1 (0-10)	0.97


**Table 4:** Characteristics of patients who conceived and who did not conceive


	Patients with endometrioma and DIE n=43	Patients with endometrioma without DIE n=61	P value

**Age (Y), median (range)**	32 (26-39)	33 (24-42)	0.4
**Patients smoking, N (%)**	11 (25.6%)	9 (14.8%)	0.26
**BMI (kg/m^2^), median (range) **	22.1 (17.9-31.1)	22.6 (17.2-35.4)	0.85
**Duration of infertility (Y), median (range)**	3 (1-9)	3 (1-8)	0.25
**Failure of IUI, N (%)**	11 (26%)	11 (18%)	0.49
**Associated tubal infertility , N (%)**	24 (55.8%)	32 (52.4%)	0.89
**Associated male infertility, N (%)**	13 (30.2%)	30 (49.1%)	0.08
**Type of ART, N (%)**			
ICSI	10 (23.2%)	26 (42.6%)	
IVF	33 (76.8%)	35 (57.4%)	
**Unilateral endometriomas, N (%) **	19 (44%)	39 (64%)	0.07
**Bilateral endometrioma, N (%)**	24 (56%)	22 (36%)	
**Size of the largest endometrioma (mm), median (range)**	34.5 (10-90)	33 (10-100)	0.84
**Associated DIE, N (%)**	22 (51.1%)	45 (73.8%)	0.03
**Patients with prior surgery for endometriosis, N (%)**	31 (72%)	41 (67%)	0.75
**AMH serum level >1 (ng/ml),median (range)**	36 (92.3%)	45 (76.3%)	0.07


**Fig 1:**
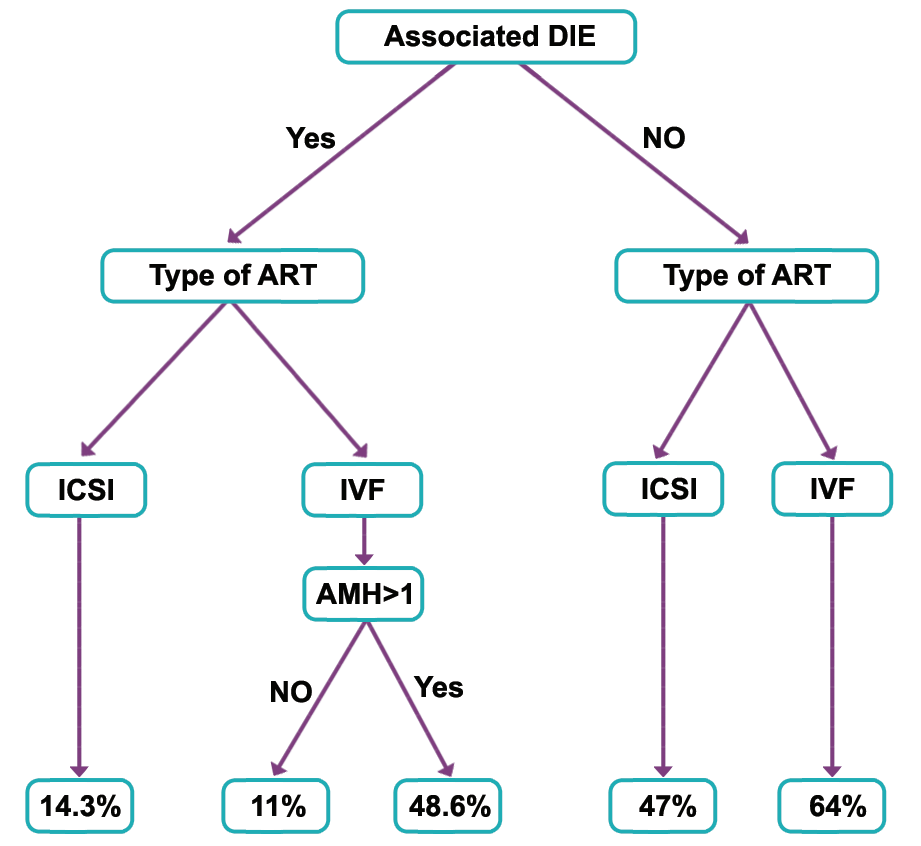
Recursive partitioning model to predict pregnancy rate.

**Fig 2:**
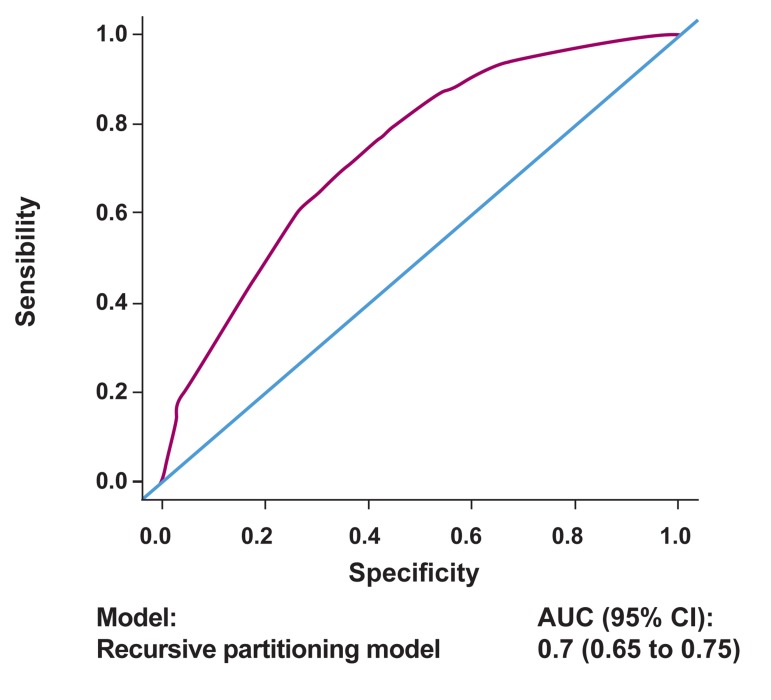
Calibration of the model with an ROC AUC.

Multivariable analysis identified three independent factors of pregnancy rate. A lower rate was associated with the presence of DIE (OR=0.24, 95% CI: 0.085-0.7, p=0.009) and the use of ICSI (OR=0.23, 95% CI: 0.07-0.8, p=0.02) and higher rate with an AMH serum level above 1 ng/ml (OR=4.3, 95% CI: 1.1- 19, p=0.049). After RP, the presence of DIE emerged as the most likely determinant factor of pregnancy (Fig 1). The calibration of the model was good with an ROC AUC (95% CI) of 0.70 (0.65-0.75) (Fig 2).

## Discussion

This study demonstrates that the presence of DIE in patients with endometrioma requiring an ICSI-IVF for infertility has a negative impact on pregnancy rate.

Indications to treat endometrioma before ICSI-IVF in infertile patients have been the source of controversy. A recent meta-analysis including four trials (7) demonstrated that laparoscopic aspiration or cystectomy of endometrioma prior to ICSI-IVF did not show evidence of benefit over expectant management on clinical pregnancy rates. However, these authors did not evaluate the impact of associated DIE on fertility outcomes. While Redwine (15) reported that isolated endometriomas were observed in less than 1.1% of patients suggesting that endometrioma management cannot be analyzed independently of the presence of other locations of endometriosis, this author did not differentiate patients with superficial peritoneal endometriosis from those with DIE. In the present study, about two-thirds of infertile women with endometrioma had associated DIE proved by clinical examination, trans-vaginal sonography and MRI. Before ICSI-IVF, the failure rate of intra-uterine insemination (IUI) was significantly higher in patients with endometrioma and DIE underlining the need to distinguish between infertile patients with endometrioma and DIE, and those without. Stepniewska et al. (10) found that removal of DIE in infertile women increased the pregnancy rate of ART including IUI and IVF but did not take into account the association of endometrioma with DIE. Pabuccu et al. (16) investigated the outcome of ICSI cycles in women with mild-to-moderate endometriosis and endometrioma but none of the patients included in the trial had DIE. Finally, only one of the four trials of the meta-analysis included patients with associated infertility factors such as male sub-fertility, cervical and tubal factor (17). This is particularly important as more than half of the patients in this study had associated tubal infertility often subsequent to anatomical distortion of the fallopian tubes linked to DIE and nearly half of the patients also had associated male infertility. Therefore, the conclusion of this meta-analysis is relevant only for the small subgroup of patients with isolated endometrioma and this is far from the reality of clinical practice. 

Using multivariable analysis, the present study has demonstrated that endometrioma associated DIE, AMH serum levels and the type of ART (IVF or ICSI) were independent prognostic factors of pregnancy. In a study comparing conservative surgery for rectovaginal endometriosis with expectant management, Vercellini et al. (3) reported a 12-month and a 24-month cumulative probability of conception of 20.5 and 44.9% respectively in the former group and 34.7 and 46.8% respectively in the latter (not significant) suggesting that excision of rectovaginal endometriosis does not improve the likelihood of pregnancy nor reduce time-toconception. In a review of the literature, these authors concluded that the purported benefit of excision of rectovaginal endometriosis in infertile patients reported by several authors may be attributed to treatment of co-existing peritoneal and ovarian endometriosis (18). These results contrast with those of other authors suggesting that the removal of DIE enhanced both spontaneous pregnancy and increased pregnancy rates in IUI and IVF treatments (10). Moreover, in a randomized trial comparing laparoscopy to open surgery for colorectal resection of endometriosis, Daraï et al. (19) found that removal of lesions enhanced spontaneous pregnancy even in patients with prior failure of IVF. Appasamy et al. (20) reported that a cumulative score using basal FSH, basal AMH, delta E2, delta inhibinB, AFC and age was the best predictor of ovarian reserve with a ROC AUC of 0.91. In the present study, among biological parameters and AFC, the sole independent factor was AMH serum level. These results are in agreement with those of Buyuk et al. (21) who reported that patients with elevated AMH serum level ≥0.6 ng/ mL had twice the number of oocytes retrieved, a greater number of day-3 embryos and a higher clinical pregnancy rate compared with patients with an AMH serum level below this value. In a logistic regression analysis, La Marca et al. (22) found that AMH and age were the only independent predictive criteria of live birth but with a sensitivity of 79.2% and a specificity of only 44.2%. A lower pregnancy rate was associated the use of ICSI. Indeed, ICSI bypasses the selective biological barrier of the zona pellucida and increases the probability of introducing an abnormal spermatozoa into the oocyte (23-25) which is detrimental to embryo development.

A few studies have focused on patient and endometriosis characteristics that may be useful to evaluate the individual probability of pregnancy in infertile patients. Younis et al. (26) recommended the use of a scoring system taking into account both epidemiological and biological characteristics and the antral follicle count to predict fertility results in IVF. However, this score does not take into account the presence of DIE which appears the most relevant predictive factor of pregnancy rate in our model. Similarly, Adamson et al. (27) recommended the use of a fertility index to evaluate the probability of obtaining spontaneous pregnancy in patients with endometriosis taking into account patient age, duration of infertility, prior pregnancy, tubal and total ASRM scores without distinguishing between patients with or without DIE. As previously mentioned, the covariates of our model are clinically significant and concordant with the published data underlining its potential use in routine practice. Thus, further studies are required to evaluate the calibration of the model, an important parameter reflecting the accuracy of prediction for continuous models by giving an idea of the model’s performance when extrapolated to a new patient population.

Some limitations of the present study have to be underlined. First, the retrospective nature of this study cannot exclude all potential biases. Secondly, the true impact of DIE in patients with infertility associated with endometrioma can only be truly assessed by a prospective trial comparing fertility results of ART in patients with DIE compared to those after removal of DIE. Third, the higher BMI in patients with endometrioma and DIE in our study constitutes a compounding factor. However, the number of obese patients (BMI >30 kg/m²) was low (2.6%) and no difference in response to hormonal ovarian stimulation was observed among the groups. Fourth, we used only the data from the first ICSI-IVF cycle of each patient to develop the model which means that the cumulative pregnancy rate after several cycles could not be evaluated. Finally, further external validation studies are required before the use of the presented model in clinical practice.

## Conclusion

The data in this study support that DIE associated with endometrioma in infertile patients has a negative impact on the pregnancy rate in first cycle ICSI-IVF. Moreover, the resultant predictive model of pregnancy rate could provide better prediction for couples about the chances of conceiving, thereby contributing to a comprehensive strategy of infertility management.
